# Bilateral posterior fracture-dislocation of the shoulders following epileptic seizures: a case report and review of the literature

**DOI:** 10.1186/s13104-015-1674-y

**Published:** 2015-11-23

**Authors:** Jagath Pushpakumara, Sivagamaroobasunthari Sivathiran, Lasantha Roshan, Saman Gunatilake

**Affiliations:** University Medical Unit, Ward 01, Colombo South Teaching Hospital, Kalubowila, Colombo, Sri Lanka; Department of Medicine, Faculty of Medical Sciences, University of Sri Jayewardenepura, and Colombo South Teaching Hospital, Colombo, Sri Lanka; Board of Study in Medicine, Postgraduate Institute of Medicine, Colombo, Sri Lanka

**Keywords:** Bilateral fracture dislocation, Shoulders, Generalized seizures

## Abstract

**Background:**

Bilateral posterior fracture-dislocation of the shoulders is an uncommon complication of grand mal seizures. We report a case of bilateral posterior dislocation of the shoulders with proximal humeral fractures following epileptic seizures. A posterior fracture-dislocation of the shoulder is very rare and can be caused by epileptic seizures, trauma, electrocution or electroconvulsive therapy.

**Case presentation:**

A 62-year-old Sri Lankan male was admitted to our medical unit following four repeated generalized tonic–clonic convulsions, each lasting for several minutes. Following the second seizure he reported an inability to move both upper arms due to intense pain. There was no history of fall during the episodes of convulsions however but the bystanders have forcibly restrained his movements during the tonic–clonic convulsions. Clinical examination revealed severely restricted range of movement in both shoulders, with associated swelling and bruising of the shoulder joints and upper arms. Radiographs of the shoulders confirmed fractures at the level of surgical neck with posterior dislocation.

**Conclusion:**

Bilateral posterior fracture-dislocation of shoulders complicating epileptic seizures are rare. Forcible restraining of the patient’s upper limbs during seizures is the likely cause for the fracture dislocations in our patient and this had not been reported before.

## Background

Bilateral fracture-dislocation of shoulder joint is a rare complication of epileptic seizures. Reports show this can be also caused by traumatic injuries and electrocution [1]. Fractured neck of humerus can also accompany simultaneous shoulder dislocation either in the anterior or posterior plane. Posterior dislocation is rare compared to anterior dislocation and is seen as a characteristic complication of seizures [2]. A literature review revealed around 30 case reports of bilateral anterior shoulder dislocations, out of which 15 were associated with simultaneous fractures [1]. Of these 15 cases most had been due to trauma or electrocution. The remainder had been attributed to epileptic or hypoglycemic seizures [1, 3]. It is said that anterior shoulder dislocations happen always secondary to trauma whereas posterior shoulder dislocations usually occur following unbalanced forceful muscle contractions that occur in conditions such as electric shock, and epileptic seizures [4, 5].

In a retrospective case control study of a total of 334 patients, the age, first episode of dislocation and the mechanism of dislocation were the three risk factors that predisposed to fractures that were associated with shoulder dislocation [6].

## Case presentation

A 62-year-old Sri Lankan male with hypertension, dyslipidemia and recent (4 months back) left sided nephrectomy due to renal cell carcinoma, was admitted to our medical unit following four episodes of generalized tonic–clonic convulsions, each lasting for several minutes. The first two episodes happened whilst the patient was at home. The second seizure had occurred several hours after the first episode on the same day while he was climbing a stair case. Relatives have restrained his jerky movements by holding both upper limbs firmly from either side to prevent him falling down the stairs. Following the second seizure he had noticed that he was unable to move his both upper limbs due to intense pain and the patient was subsequently admitted to hospital for further management. During the hospital stay he developed two more generalized seizures while during sleep, 24 h apart. There was no history of fever, headache, vomiting or altered behavior suggestive of meningo-encephalitis. There was no aura prior to seizures. He reported no history of visual disturbances, unsteadiness, alcohol or substance abuse. The relatives assured that he did not have any falls or mechanical trauma during convulsions. Clinical examination revealed severely restricted bilateral shoulder movements, swelling of both shoulder joints with marked bruising involving upper limbs and posterior upper chest. The patient had severe pain with the slightest movement of the shoulders. There was no papilledema or neck rigidity and the neurological examination was normal. His blood pressure was 130/80 mmHg and pulse rate 80 beats per minute. Respiratory and abdominal examination was normal.

**Fig. 1:**
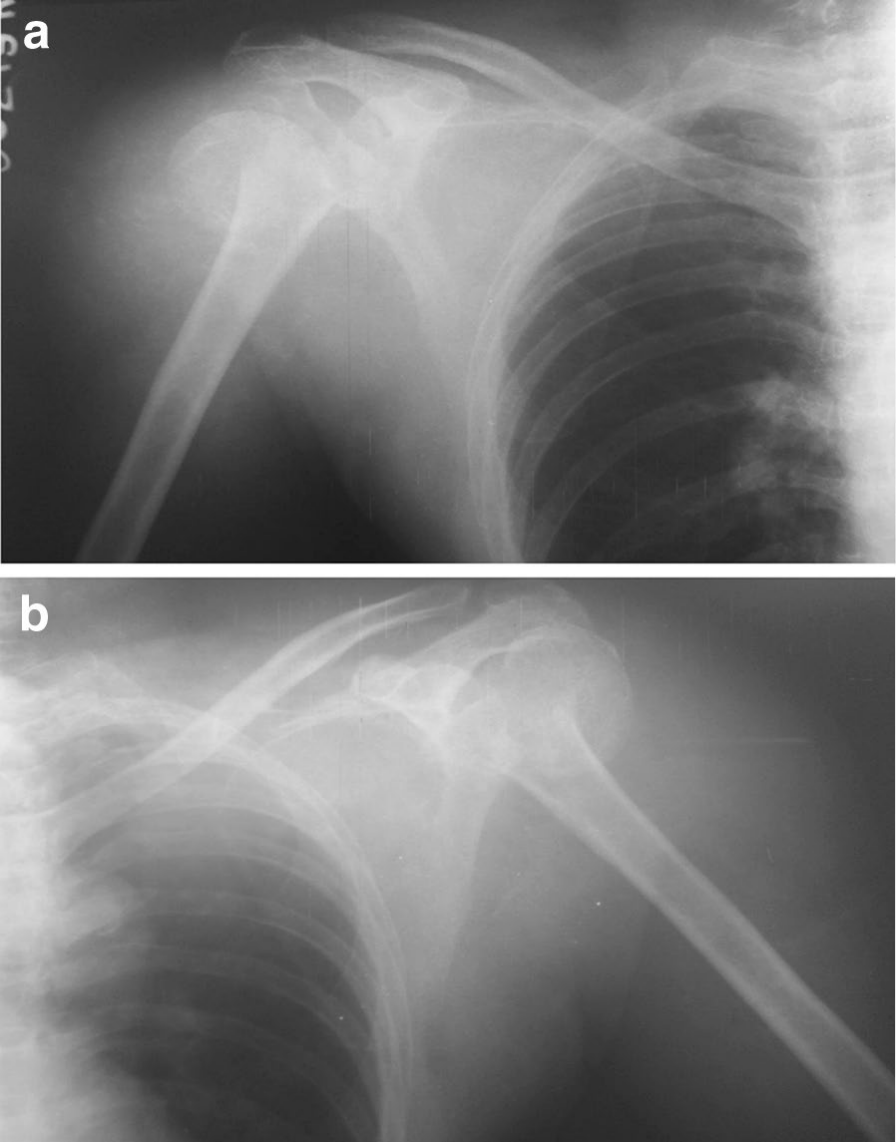
Fracture with posterior dislocation. **a** Right shoulder, **b** left shoulder

Shoulder radiographs revealed fractures at surgical neck of humerus with posterior shoulder dislocation in both shoulders (Fig. 1 a, b). His initial hemoglobin was 11.1 g/dl and rapidly dropped to 6.3 g/dl over 48 h due to bleeding into upper limb muscles and subcutaneous tissues following fractures. The white cell and platelet counts were within normal limits. He had evidence of rhabdomyolysis evidenced by very high creatinine phosphokinase (CPK) of 38711 U/l (normal < 120). This gradually declined to 1514 U/l over 1 week. His basic biochemical investigations were; sodium 132 mmol/l, potassium 3.4 mmol/l, serum calcium 2.55 mmol/l (2.15–2.5), serum magnesium 1.23 mmol/l (0.33–1.3) and serum creatinine 148 µmol/l which normalized to 65 µmol/l after a week.

Magnetic resonance imaging (MRI) of brain did not show metastatic deposits from the previously treated left sided renal cell carcinoma or other epileptogenic focus. Radiological survey did not reveal any other bone metastasis of renal cell carcinoma. His electroencephalogram (EEG) done 24 h after the seizures was normal. He was diagnosed to have idiopathic epilepsy based on the clinical history, examination and investigations.

He was transfused with five pints of blood. His renal functions and CPK were monitored daily. He was hydrated well with intravenous normal saline to prevent myoglobinuria induced acute kidney injury during the initial stage. His pain was managed with regular analgesics. He was started on phenytoin sodium 100 mg twice daily. The patient was assessed by the consultant orthopedic surgeon and decided to manage with close reduction and conservative measures. Bilateral collar and cuff with body strapping were applied. Six months after the injury his shoulder movements were satisfactory.

## Discussion

Bilateral posterior fracture-dislocation of the shoulder, first described in 1902 by Mynter [7] is extremely rare. The most common cause for this situation is a convulsive seizure [8]. Of 2800 patients admitted to hospital with a diagnosis of seizure, 1.1 % (30/2800) sustained fractures [9]. Of these it was found that 0.5 % (15/2800) had fractures due to direct trauma, 0.3 % (7/2800) had sustained a fracture as a complication of seizure alone and 0.3 % (8/2800) sustained a fracture for which the etiology was not determined. In the trauma group 11 out of 17 fractures involved clavicle, nasal bone and the skull while in non-trauma group six of nine fractures occurred in the proximal humerus.

The proposed mechanism of shoulder injury during a seizure is well described [10]. The typical position of the shoulder during a convulsion is adduction, internal rotation and flexion. When the seizure continues humeral head is forced superiorly and posteriorly over the glenoid cavity, and when it stops, the humeral head stays locked behind the glenoid cavity. Further convulsions cause humeral head to be impinged against the glenoid rim resulting in eventually a complex proximal humeral fracture.

Fracture dislocation of shoulder can solely be due to epileptic seizure or trauma which occur during a convulsion. The latter could be due to direct impact on nearby objects or due to falls. In addition to those, forceful restraining of limbs during seizures may give rise to fracture or dislocation as in our patient. Untrained non-medical personnel tend to restrain the patients while they are having epileptic seizures, which can give rise to traumatic injuries. Some people insert objects into the patient’s mouth to prevent tongue biting during convulsions and this can cause traumatic injury to the oral cavity and displacement of teeth. Forceful limb restrain while having a convulsion can give rise to fractures and dislocation of joints especially shoulder and hip joints.

There were two case reports published in 2009, of those, one is a 36-year-old man with a dominant right hand, a farm worker, with a history of alcohol abuse and the other one was a 57-year-old housemaid with a dominant right hand. Both patients developed bilateral posterior fracture-dislocation of shoulders the first following electrocution and the second following a major convulsive episode [11].

The diagnosis of the fracture-dislocation of shoulders occurring due to a seizure is often delayed and up to 50 % are not diagnosed in the first hospital visit [12]. In a patient presenting with upper limb pain and reduced movements following seizures, fracture dislocation should be suspected and appropriate imaging should be done. Anterior bruising of the upper arm is a useful clinical feature seen often. Routine radiological assessment is done with antero-posterior and axillary views of shoulders, but when surgical treatment is considered computed tomography (CT) images of shoulders are needed as they provide more information such as better visualization of the trough fracture in the humeral head than conventional X-ray films. It also demonstrates fracture fragments and their alignment which may not be seen on conventional X-rays [12].

Epilepsy, electrocution and extreme trauma (also known as triple E syndrome) are the causes of fracture-dislocation of shoulders [7]. Almost 50 % of bilateral posterior dislocations are due to epileptic seizures, and when associated with a fracture this rises to about 90 %. The diagnosis of epilepsy is based on proper history obtained from an eye witness and supported by brain imaging and EEG. Our patient’s brain MRI and EEG both were normal. But EEG has relatively low sensitivity in epilepsy, ranging between 25 and 56 %. Specificity is better but again variable at 78–98 % [13]. Electrocution accounts for less than 5 % of bilateral posterior dislocations of the shoulders [11]. The common complications of shoulder fracture dislocation are avascular necrosis of the humeral head, secondary osteoarthritis and impaired range of movements.

Management of fracture-dislocation of the shoulder depends on several factors such as the type of fracture, severity of injury, whether unilateral or bilateral and time taken to diagnose. The most simple but comprehensive classification is the Neer’s classification which was based on careful analysis of radiographs and surgical findings from 300 proximal humeral fractures treated at the New York Orthopedic Hospital-Columbia Presbyterian Medical Center between 1953 and 1967 [14].

In many cases reported, the fracture was a large compression defect in the antero-medial aspect of the articular surface of the humeral head. When the shoulder images show minimal displacement of fragments and the viability of the humeral head is not in doubt close reduction should be done. It has been suggested that for defects which involve less than 20 % of the articular surface, close reduction can be attempted [15]. Rush nails or percutaneous K wires can be used to maintain reduction. Open reduction is necessary for defects that involve 20–40 % of the surface. It has been stated that 3 weeks after trauma, closed reduction is almost impossible and surgical treatment is required [16]. Young patients who have acute displaced fractures, gentle closed reduction can be attempted, and when it is not successful, open reduction and internal fixation is the option. If open reduction cannot be achieved or in cases in which more than 50 % of the articular surface of the head is damaged, hemiarthroplasty is the choice. In older patients’ (age > 65 years) when there is three or four part fracture, there is high risk of avascular necrosis of the humeral head. Because of this reason the better treatment option is hemiarthroplasty. When both humeral head and glenoid cavity are damaged, a total shoulder arthroplasty may be considered [17]. Therefore the treatment should be decided depending on the type of lesion, the interval of time between trauma and treatment, age of the patient and occupation [18].

**Fig. 2:**
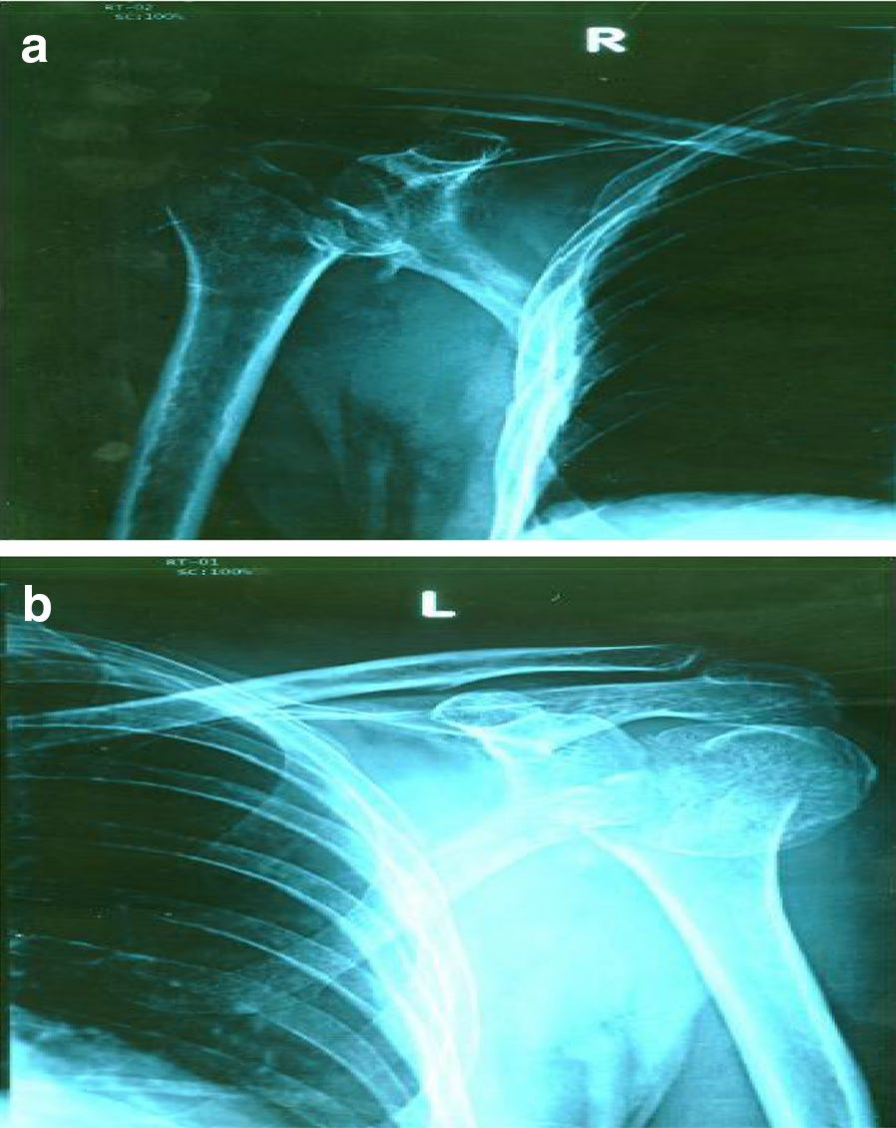
Shoulder X-rays done after 6 weeks showing evidence of healing. **a** Right shoulder, **b** left shoulder

**Fig. 3:**
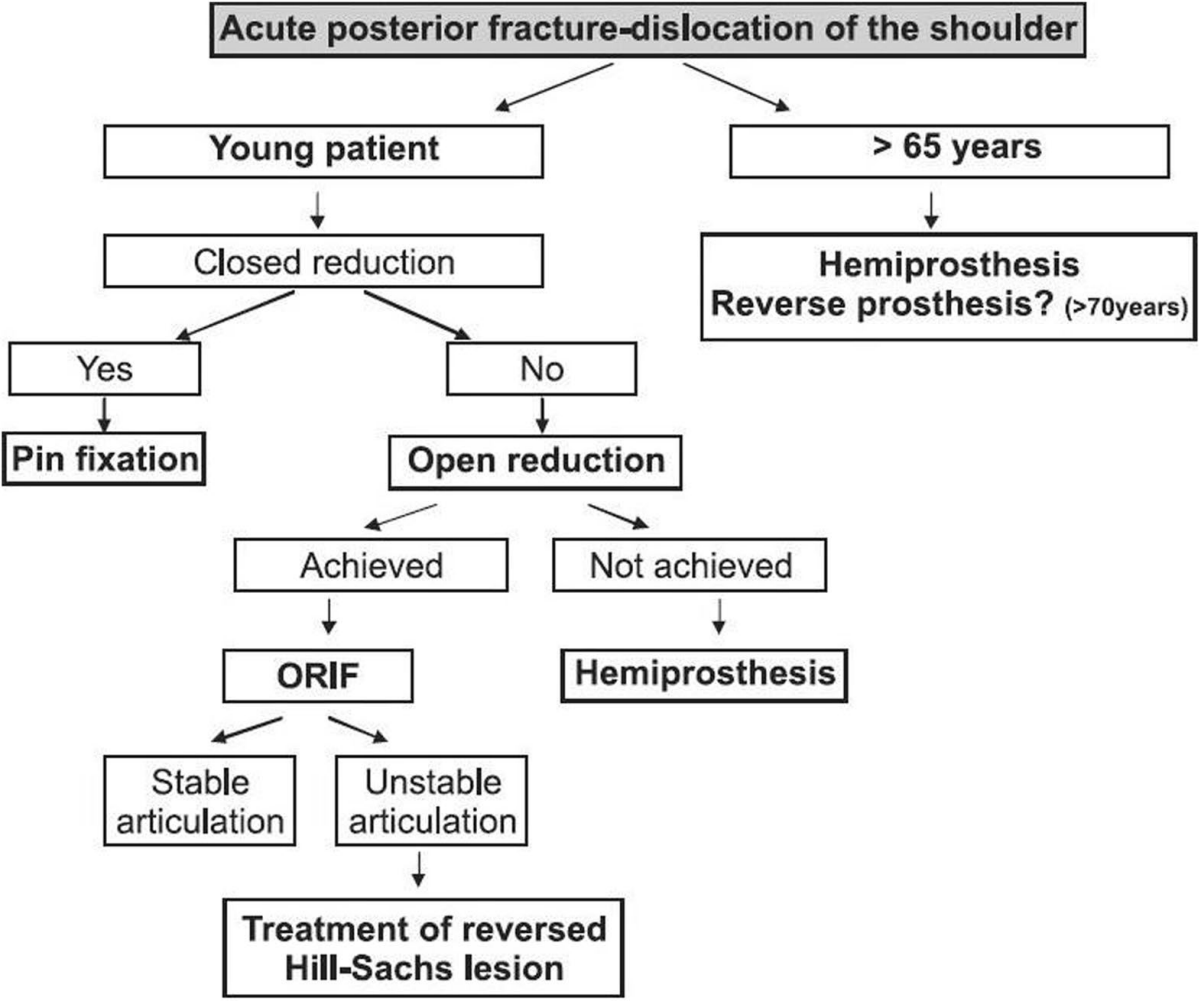
Management algorithm of acute posterior fracture dislocation of the shoulder

Our patient had bilateral two part fracture at the level of surgical neck of humerus with posterior dislocation of humeral head. He was managed with color and cuff sling with body strapping applied to both upper limbs with regular clinic follow up by the orthopedic team. His follow up X-rays of shoulders, taken after 6 weeks showed evidence of healing (Fig. 2 a, b). Movement of his shoulders and pain improved with this treatment. The management algorithm of acute posterior fracture-dislocation of shoulder is shown in Fig. 3 [11].

## Conclusion

We report a rare case of bilateral posterior fracture-dislocation of the shoulders as a complication of epileptic seizures. Possible secondary causes for fractures were excluded. Bilateral posterior fracture-dislocation of shoulders complicating epileptic seizures are rare. Forcible restraining of the patient’s upper limbs during seizures is the likely cause for the fracture dislocations in our patient and this had not been reported before.

## Consent

Written informed consent was obtained from the patient for publication of this case report and any accompanying images.

## Abbreviations

CPK: creatinine phosphokinase
MRI: magnetic resonance imaging
EEG: electroencephalogram
CT: computed tomography
